# Higher Caffeinated Coffee Intake Is Associated with Reduced Malignant Melanoma Risk: A Meta-Analysis Study

**DOI:** 10.1371/journal.pone.0147056

**Published:** 2016-01-27

**Authors:** Jibin Liu, Biao Shen, Minxin Shi, Jing Cai

**Affiliations:** Nantong Tumor Hospital, Nantong Pingchao town, Tong yang Rd. 30, 226361, Jiangsu province, China; University of Modena & Reggio Emilia, ITALY

## Abstract

**Background:**

Several epidemiological studies have determined the associations between coffee intake level and skin cancer risk; however, the results were not yet conclusive. Herein, we conducted a systematic review and meta-analysis of the cohort and case-control studies for the association between coffee intake level and malignant melanoma (MM) risk.

**Methods:**

Studies were identified through searching the PubMed and MEDLINE databases (to November, 2015). Study-specific risk estimates were pooled under the random-effects model.

**Results:**

Two case-control studies (846 MM patients and 843 controls) and five cohort studies (including 844,246 participants and 5,737 MM cases) were identified. For caffeinated coffee, the pooled relative risk (RR) of MM was 0.81 [95% confidential interval (95% CI) = 0.68–0.97; P-value for Q-test = 0.003; I^2^ = 63.5%] for those with highest versus lowest quantity of intake. In the dose-response analysis, the RR of MM was 0.955 (95% CI = 0.912–0.999) for per 1 cup/day increment of caffeinated coffee consumption and linearity dose-response association was found (P-value for nonlinearity = 0.326). Strikingly, no significant association was found between the decaffeinated coffee intake level and MM risk (pooled RR = 0.92, 95% CI = 0.81–1.05; P-value for Q-test = 0.967; I^2^ = 0%; highest versus lowest quantity of intake).

**Conclusions:**

This meta-analysis suggested that caffeinated coffee might have chemo-preventive effects against MM but not decaffeinated coffee. However, larger prospective studies and the intervention studies are warranted to confirm these findings.

## Introduction

Malignant melanoma (MM) is a lethal type of skin cancer, and its incidence has been increasing worldwide for the past several years [1]. Many factors have been identified that may influence the susceptibility of people to MM including ultraviolet (UV)-light exposure, skin phototype, eye and hair color, tanning capacity, presence of freckles and/or precancerous lesions, larger number of common acquired nevi, presence of atypical nevi, and genetic factors [2]. Solar ultraviolet was recognized as one of the most notable environmental factors for MM risk, although many lifestyle and environmental factors influence MM risk through interaction with UV exposure. Several dietary or nutritional factors, including the amount of ingestion of fish, vegetables and fruits, were suggested to have preventative effects against melanoma and a few studies have reported protective roles of beta-carotene and vitamins A, C, D and E on melanoma. However, these identified susceptibility factors only account for a small proportion of melanoma risk [3]. Caffeine, an ingredient found at high concentrations in coffee, has been reported to inhibit UV-induced skin carcinogenesis. Oral administration of caffeine could enhance UV-induced cell apoptosis [4], promote the elimination of DNA damaged cells [4], reduce the inflammation of epidermal cells [5] and inhibit the aberrant DNA methylation of the cells [6]. Moreover, caffeine also inhibits the metastasis of melanoma tumor cells [7] and enhances the radiosensitivity of tumor cells [8]. Therefore, higher coffee intake may have preventative effects against cutaneous melanoma. To date, several epidemiological studies have determined associations between caffeinated coffee intake level and the risk for MM and found that caffeinated coffee may have protective effects against melanoma [9–11]. However, the results for other studies found no such associations [12–14]. In contrast, several epidemiological studies have also evaluated the associations between the decaffeinated coffee and melanoma risk, but none of them found a statistically significant association [9,12,13,15–18]. As the results of the epidemiological studies are inconsistent, we herein systematically reviewed the epidemiological studies that have assessed the associations between the caffeinated or decaffeinated coffee intake level and the risks for MM, and further assessed their associations utilizing meta-analysis methods.

## Methods and Materials

### Eligibility study identification

**Fig 1:**
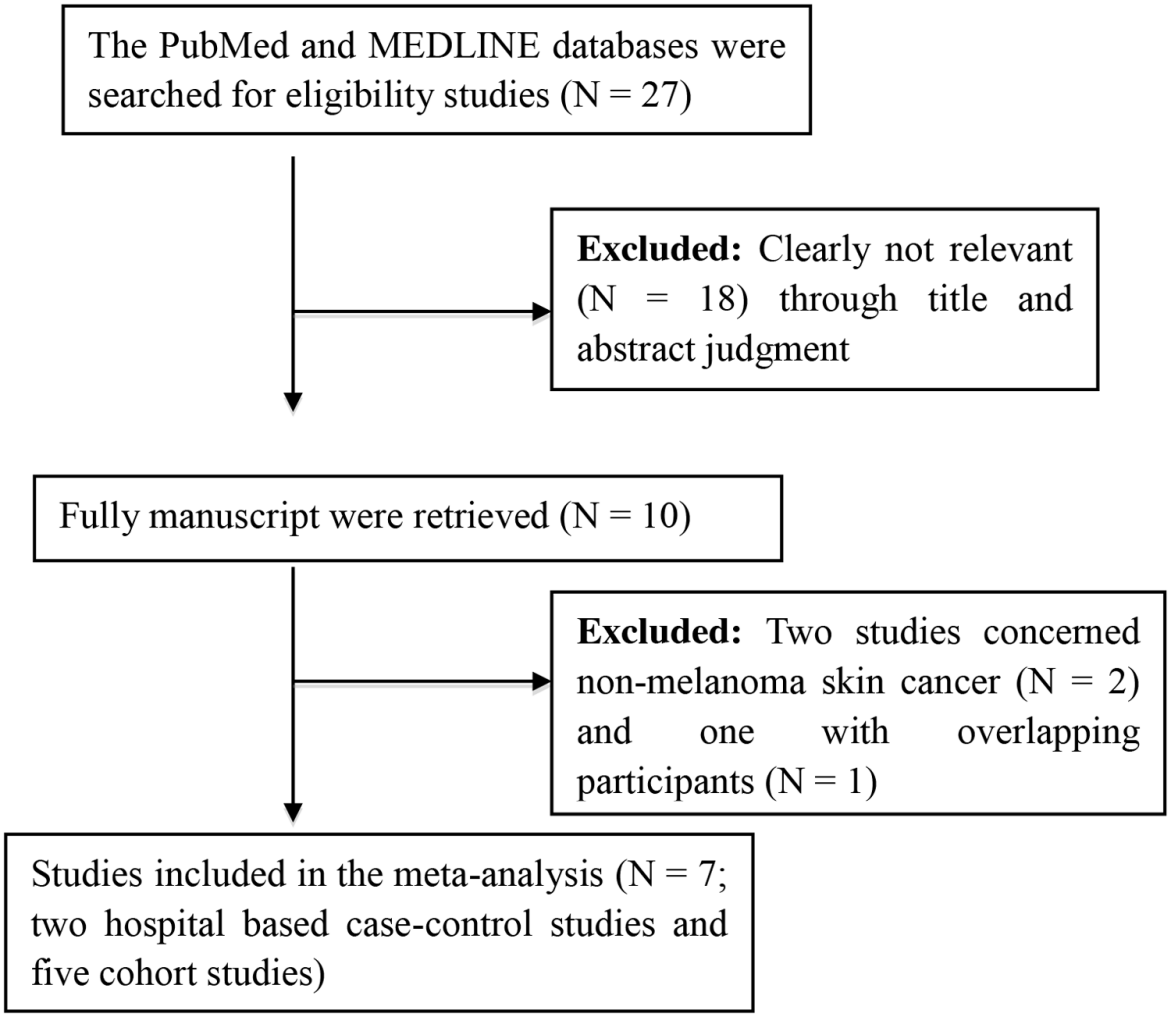
The working flow chart for eligible study identification in meta-analysis studies.

To identify the eligibility studies that have determined the association between the coffee intake level and the risk for MM, two authors (Liu and Shen) independently searched the PubMED and MEDLINE databases with the terms “melanoma” or”skin cancer” in combination with “coffee” or “caffeine”. The electronic search was performed in November, 2015 and the eligibility studies should be online published online in peer-reviewed journals. The titles, abstracts and the full manuscripts were independently reviewed and assessed by the two authors, and the third author (Cai) joined the assessment of the manuscript if there was any discrepancy. In addition, we further checked the references of the retrieved studies and the published reviews or comments to identify any missing study in the literature search. In brief, the working flow chart of the eligible literature identified was presented as Fig 1.

### Inclusion and exclusion criteria

Epidemiological studies that were to be included in the current meta-analysis studies should meet the following criteria: 1) was a population- or hospital-based, case-control study or a prospective or retrospective cohort study; 2) provided the coffee intake level category and the corresponding relative risk (RR) or the odds ratio (OR) estimates and their confidential intervals (95% CIs) for MM; 3) not overlapped participants with other included studies. If overlapping populations were identified between studies, the most complete one was included in the meta-analysis; 4) be reported in the English language. Here, we excluded conference abstracts, reviews, ecological studies, cellular studies and animal studies. Studies that concerned the non-melanoma skin cancer were also excluded.

### Quality assessment of the included studies

We assessed the quality of the evidence, for each study, on the association between caffeinated or decaffeinated coffee intake level and melanoma risk using the Newcastle-Ottawa quality scale [19]. For case-control or cohort studies, a total of nine points were designated based on the characteristics of the studies including: (1) the selection of comparison groups, total score: 4; (2) the comparability of the groups, total score: 2; (3) the quality of the measurement of exposure and outcome, total score: 3. The total score of the adapted studies ranged between 5 to 7, and we deemed those with a score of 6 or lower as poorer quality studies (S1 Table).

### Data extraction

The following data was extracted from each study: the last name of the first author, the publication year, the study design (hospital or population based case-control studies or the cohort studies), the country where the study was performed, the sample size (the number of participants for cohort studies and the number of patients and controls for case-control studies), the study period for the cohort studies (the year for the participants recruitment and the last following-up year), the types of coffee (caffeinated or decaffeinated), the method for the exposure assessment, the intake category for the coffee intake level and the corresponding relative risk (RR) or odds ratio (OR) estimates with their 95% confidential intervals (95% CIs), and the adjustments in the statistical analyses. If there were subgroup studies, each subgroup was recognized as individual studies. The meta-analysis studies were performed following the MOOSE (S1 File) and PRISMA guidelines (S2 File).

### Statistical analysis

The inverse variance weighting method was applied to calculate the summary estimates and the 95% CIs. The risk estimates for the highest, in comparison with the lowest, category for each study were extracted or calculated, and the squared inverse variance for the logarithm estimate was considered as the appropriate weight for each study. The ORs from case-control studies were assumed as the estimates of the RRs for prospective studies. The DerSimonian and Laird random-effects model that considers both within- and between-study heterogeneity were used in the pooling analyses [20]. The Cochran's Q-test in together with the I^2^ statistic were used to assess the heterogeneity between the studies. Significant heterogeneity was considered when P < 0.05 for Q-test or the I^2^ was larger than 25%. To identify any individual study that may significantly affect the pooled estimates, the sensitivity analyses were performed through calculating the pooled risk estimates after excluding each single study from the meta-analysis repeatedly. The publication bias for the studies was assessed with the Begg’s funnel plots and further determined with the Egger’s linear regression test [21,22]. To investigate the influence of the basic characteristics of the individual studies on the heterogeneity between the results of multiple studies, the meta-regression analyses were performed. We also applied the generalized least square estimated trend (GLST) analysis methods proposed by Orsini et al. [23] to evaluate the dose-response relationship between coffee intake and MM risk in the prospective cohort studies. The restricted cubic spline methods with three knots at 10%, 50% and 90% for the distribution of daily caffeinated coffee intake were used to evaluate the linear relationship between coffee intake and MM risk. All statistical analyses were performed with R (www.r-project.org), Stata (version 11.0, StataCorp) and Review Manager (version 5.2.10) software. Two-sided P < 0.05 was considered to be statistically significant.

## Results

### Literature search and study characteristics

In the literature search, we have identified seven articles, reported from two case-control studies [10,16] and five cohort studies that have determined the association between the coffee intake level and the MM risk [9,11–14]. The basic characteristics of the included studies are presented in Table 1. The two case-control studies, both of which were conducted in Italy, have recruited a total of 846 MM patients and 843 controls. Five prospective cohort reports with eight subgroups involved a total of 844,246 participants and 5,737 cases. Of them, three were performed in the United States and one in Norway (Table 1). According to the Newcastle-Ottawa quality scale guidelines, three studies were designated as being conducted with poorer quality and three studies were conducted with better quality (S1 Table).

**Table 1:** The basic characteristics for the eligibility studies included in the meta-analysis studies.

**First author, Year (Ref)**	**Study design**	**Region**	**Participants**	**Subgroup, exposure assessment**	**Intake category**	**OR/RR (95% CI)**	**Adjustments**
**Naldi L, 2004 (16)**	Hospital based case-control	Italy	542 cutaneous malignant melanoma cases and 538 controls	Caffeinated coffee, structured questionnaire	<1 cup per day/1 cup per day/2 cups per day/3 cups per day/≥4 cups per day	Referent/1.26 (0.85–1.87)/1.14 (0.77–1.67)/1.29 (0.81–2.04)/1.15 (0.68–1.92)	Age, sex, education, BMI, history of sunburns, propensity to sunburns, number of naevi, number of freckles, skin, hair and eye colour, and smoking.
				Decaffeinated coffee, structured questionnaire	Non-drinkers/Drinkers	Referent/0.84 (0.60–1.18)	The same as above.
**Fortes C, 2013 (10)**	Hospital based case-control	Italy	304 melanoma cases and 305 controls	Caffeinated coffee, structured questionnaire	≤1 cup per day/> 1 cup/day	Referent/0.53 (0.38–0.74)	Age and sex.
**Veierod M, 1997 (11)**	Prospective cohort	Norway	25,049 women (47 cases) and 25,708 men (61 cases) (1974–1976 to 1977–1983)	Caffeinated coffee in women, FFQ	≤ 2 cups per day/3-4 cups per day/5-6 cups per day/≥ 7 cups per day	Referent/0.6 (0.3–1.1)/0.4 (0.2–0.8)/0.4 (0.2–0.8)/0.4 (0.2–0.9)	County of residence, age at inclusion and attained age.
				Caffeinated coffee in men, FFQ	≤ 2 cups per day/3-4 cups per day/5-6 cups per day/≥7 cups per day	Referent/2.4 (0.8–7.1)/1.9 (0.6–5.7)/1.5 (0.5–4.6)	County of residence, age at inclusion and attained age.
**Nilson L, 2010 (14)**	Prospective cohort	Sweden	76,338 residues, 108 cases (1992–2007)	Caffeinated coffee, FFQ	< 1 cups per day/1-3 cups per day/≥7 cups per day	Referent/0.91 (0.48–1.70)/0.97 (0.50–1.89)	Age, sex, BMI, smoking, education, and recreational physical activity.
**First author, Year (Ref)**	**Study design**	**Region**	**Participants**	**Subgroup, exposure assessment**	**Intake category**	**OR/RR (95% CI)**	**Adjustments**
**Wu H, 2015 (12)**	Prospective cohort (Women's Health Initiative-Observational Study, WHI-OS)	USA	66,484 postmenopausal women, 363 cases (1993–1998 to September 2005)	Caffeinated coffee, NA	< 1 cup per day/1 cup per day/2-3 cups per day/> 4 cups per day	Referent/0.85 (0.60–1.19)/0.96 (0.73–1.26)/1.01 (0.67–1.53)	Age, height, waist-hip ratio, education, income, alcohol, smoking, region of residence, aspirin, history of nonmelanoma skin cancer, kin reaction to sun, sunscreen use, and summer sunlight exposure in the 30s.
				Decaffeinated coffee, NA	< 1 cup per day/1 cup per day/ 2–3 cups per day/> 4 cups per day	Referent/0.77 (0.55–1.09)/1.00 (0.73–1.38)/0.73 (0.36–1.49)	The same as above.
**Wu S, 2015 (9)**	Prospective cohort (Nurses' Health Study II, NHSII)	USA	89,220 women, 642 cases (1991–2009)	Caffeinated coffee, FFQ	Never/< 1 cup per day/1-2 cups per day/>2 cups per day	Referent/0.80 (0.63–1.0)/0.79 (0.62–1.0)/0.70 (0.55–0.89)	Age, family history, personal history of non-skin cancer, hair color, number of moles on legs or arms, sunburn reaction as a child/adolescent, number of blistering sunburns, time spent in direct sunlight since high school, cumulative ultraviolet flux since baseline, BMI, smoking, physical activity, total energy intake, alcohol intake, rotating night shifts, menopausal status, postmenopausal hormone use, consumption of decaffeinated coffee, caffeinated tea, decaffeinated tea, caffeinated carbonated beverages, decaffeinated carbonated beverages, and caffeine-containing chocolate.
				Decaffeinated coffee, FFQ	Never/< 1 cup per day/1-2 cups per day/>2 cups per day	Referent/0.96 (0.79–1.2)/1.10 (0.8–1.4)/0.93 (0.60–1.4)	The same as above.
**First author, Year (Ref)**	**Study design**	**Region**	**Participants**	**Subgroup, exposure assessment**	**Intake category**	**OR/RR (95% CI)**	**Adjustments**
	Prospective cohort (Nurses' Health Study, NHS)	USA	74,666 women, 841 cases (1980–2008)	Caffeinated coffee, FFQ	Never/< 1 cup per day/1-2 cups per day/>2 cups per day	Referent/0.92 (0.72–1.2)/0.86 (0.68–1.1)/0.81 (0.65–1.0)	The same as above.
				Decaffeinated coffee, FFQ	Never/< 1 cup per day/1-2 cups per day/>2 cups per day	Referent/1.20 (0.94–1.4)/1.10 (0.84–1.4)/0.98 (0.72–1.3)	The same as above.
	Prospective cohort (Health Professionals Follow-up Study, HPFS)	USA	39,424 men, 771 cases (1986–2008)	Caffeinated coffee, FFQ	Never/< 1 cupper day /1-2 cups per day/>2 cups per day	Referent/1.00 (0.83–1.3)/1.10 (0.84–1.3)/1.10 (0.86–1.3)	The same as above.
				Decaffeinated coffee, FFQ	Never/< 1 cupper day /1-2 cups per day/>2 cups per day	Referent/1.10 (0.92–1.3)/1.10 (0.91–1.4)/0.92 (0.68–1.2)	The same as above.
**Loftfied E, 2015 (13)**	Prospective (NIH-AARP Diet and Health Study, NIH-AARP)	USA	447,357 non-Hispanic whites, 2904 melanoma (1995–2006)	Caffeinated coffee, FFQ	None/≤1 cup per day/2-3 cups per day/≥ 4 cups per day	Referent/0.89 (0.77–1.03)/0.91 (0.80–1.04)/0.75 (0.64–0.89)	Age, sex, cigarette smoking and smoking intensity, cigar/pipe smoking, BMI, education, average daily alcohol intake, physical activity, family history of cancer, and July erythemal exposure.
				Decaffeinated coffee, FFQ	None/≤1 cup per day/2-3 cups per day/≥ 4 cups per day	Referent/0.92 (0.79–1.06)/ 0.90 (0.77–1.05)/ 0.95 (0.76–1.18)	The same as above.

**Abbreviations:** 95% CI, 95% confidential interval; BMI, body mass index; FFQ, food frequency questionnaire; NA, not applicable; OR, odds ratio; RR, relative risk.

### Caffeinated coffee and MM risk

**Fig 2:**
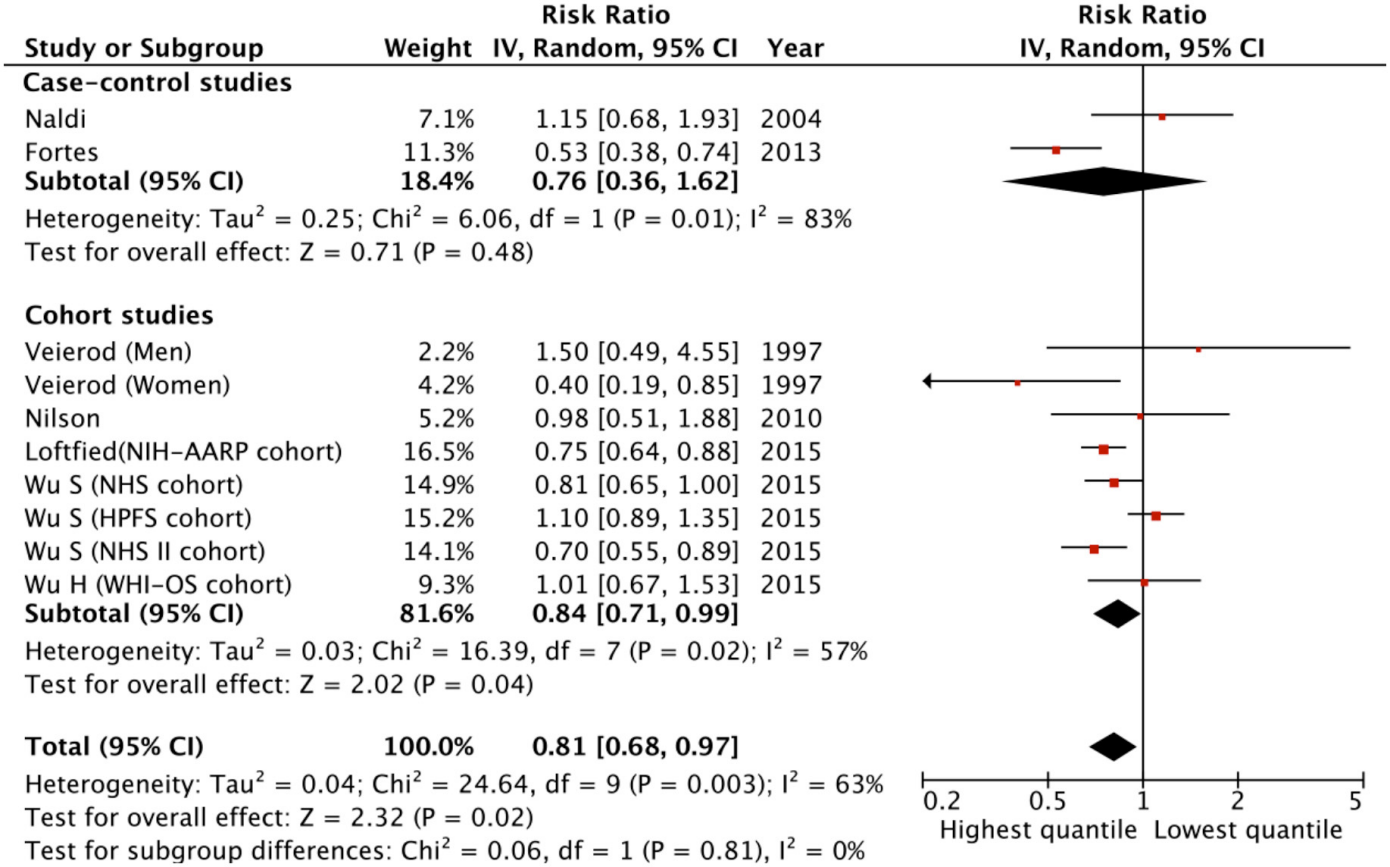
The relative risks (RRs) of melanoma for the highest versus lowest quantity of caffeinated coffee intake in case-control studies and cohort studies. The square represents the study-specific RR and the size of the squares reflects the statistical weight in the meta-analysis; horizontal lines represent the 95% confidential intervals (95% CIs); the diamond indicates the overall RR with its 95% CI under the random-effects model.

These seven articles included 10 individual subgroups have determined the association between the caffeinated coffee intake level and the risk for MM. The pooled RR was 0.81 (95% CI = 0.68–0.97) for those participants with the highest category of caffeinated coffee intake versus lowest category (Fig 2). Significant heterogeneity between the studies was noticed (Q = 24.64, df = 9, P = 0.003; I^2^ = 63.5%). However, no individual study significantly affected the pooled estimate as suggested by the sensitivity analyses. The funnel plot suggested there was no potential publication bias for the included studies (S1 Fig), which was validated with the Egger’s test (P = 0.897).

The meta-regression analyses suggested that none of the study-level characteristics (quality score, study design, gender of the participants, median or average follow-up time, study region, the lower boundary level of the highest and lowest categorized coffee intake level) significantly influence the results of multiple studies (S2 Table). In the stratification studies (Table 2), we found the association between the caffeinated coffee intake level and the MM risk was more prominent in the cohort studies (pooled RR = 0.84, 95% CI = 0.71–0.99) than in the case-control studies (pooled RR = 0.76, 95% CI = 0.36–1.62). The protective effects for the caffeinated coffee against melanoma were statistically significant in women (pooled RR = 0.76, 95% CI = 0.61–0.95) but not in men (pooled RR = 1.11, 95% CI = 0.91–1.36).

**Table 2:** The stratification studies for the associations between caffeinated coffee or decaffeinated coffee intake level (highest vs. lowest percentile) and the risk for melanoma.

Coffee Type	Subgroup category	Subgroup title	No. of subgroups	Pooled RR (95% CI)	Q	df	P-value	I^2^	Egger's test (P-value)
**Caffeinated coffee**	Overall		10	0.81 (0.68–0.97)	24.64	9	0.003	63.5%	0.897
	Study Type	Case-control	2	0.76 (0.36–1.62)	6.06	1	0.014	83.5%	-
		Cohort	8	0.84 (0.71–0.99)	16.39	7	0.022	57.3%	0.876
	Gender	Women	4	0.76 (0.61–0.95)	5.39	3	0.145	44.3%	0.630
		Both	4	0.76 (0.57–1.02)	7.36	3	0.061	59.2%	0.699
		Men	2	1.11 (0.91–1.36)	0.29	1	0.59	0%	-
	Study region	USA	5	0.85 (0.72–1.01)	11.42	4	0.022	65.0%	0.639
		Other	5	0.77 (0.49–1.20)	11.14	4	0.025	64.1%	0.409
	Study quality	Higher	7	0.82 (0.68–0.99)	15.49	6	0.017	61.3%	0.983
		Lower	3	0.83 (0.50–1.37)	8.76	2	0.013	77.2%	0.268
**Decaffeinated coffee**	Overall		6	0.92 (0.81–1.05)	0.94	5	0.967	0%	0.109
	Study Type	Case-control	-	-	-	-	-	-	-
		Cohort	5	0.94 (0.82–1.08)	0.60	4	0.964	0%	0.116
	Gender	Women	3	0.94 (0.74–1.18)	0.56	2	0.754	0%	0.125
		Men	-	-	-	-	-	-	-
	Study region	USA	5	0.94 (0.82–1.08)	0.60	4	0.964	0%	0.116
		Other	-	-	-	-	-	-	-
	Study quality	Higher	4	0.95 (0.82–1.09)	0.1	3	0.992	0%	0.804
		Lower	2	0.82 (0.60–1.11)	0.12	1	0.727	0%	-

**Fig 3:**
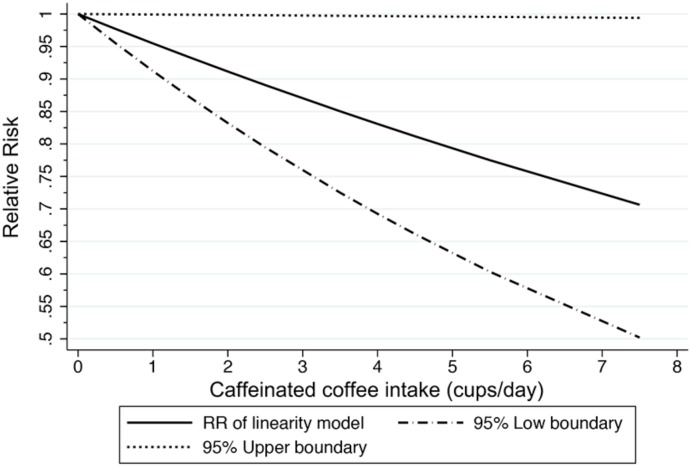
The dose-relationship between the caffeinated coffee intake (cups/day) and the MM risk compared to non-drinkers (P for non-linearity = 0.326).

To evaluate the dose-response relationship between the caffeinated coffee intake and risk for MM, five articles with eight prospective cohort studies were included in the generalized least squares trend estimation analysis [9,11–14]. We found a linear dose-response association between the caffeinated coffee intake level and MM risk (P for nonlinearity = 0.326; Fig 3). In the dose-response analysis, a 4.5% reduced MM risk (pooled RR = 0.955, 95% CI = 0.912–0.999) was noticed for per 1 cup/day increment of caffeinated coffee intake compared to non-drinkers (Fig 3).

### Decaffeinated coffee and MM risk

**Fig 4:**
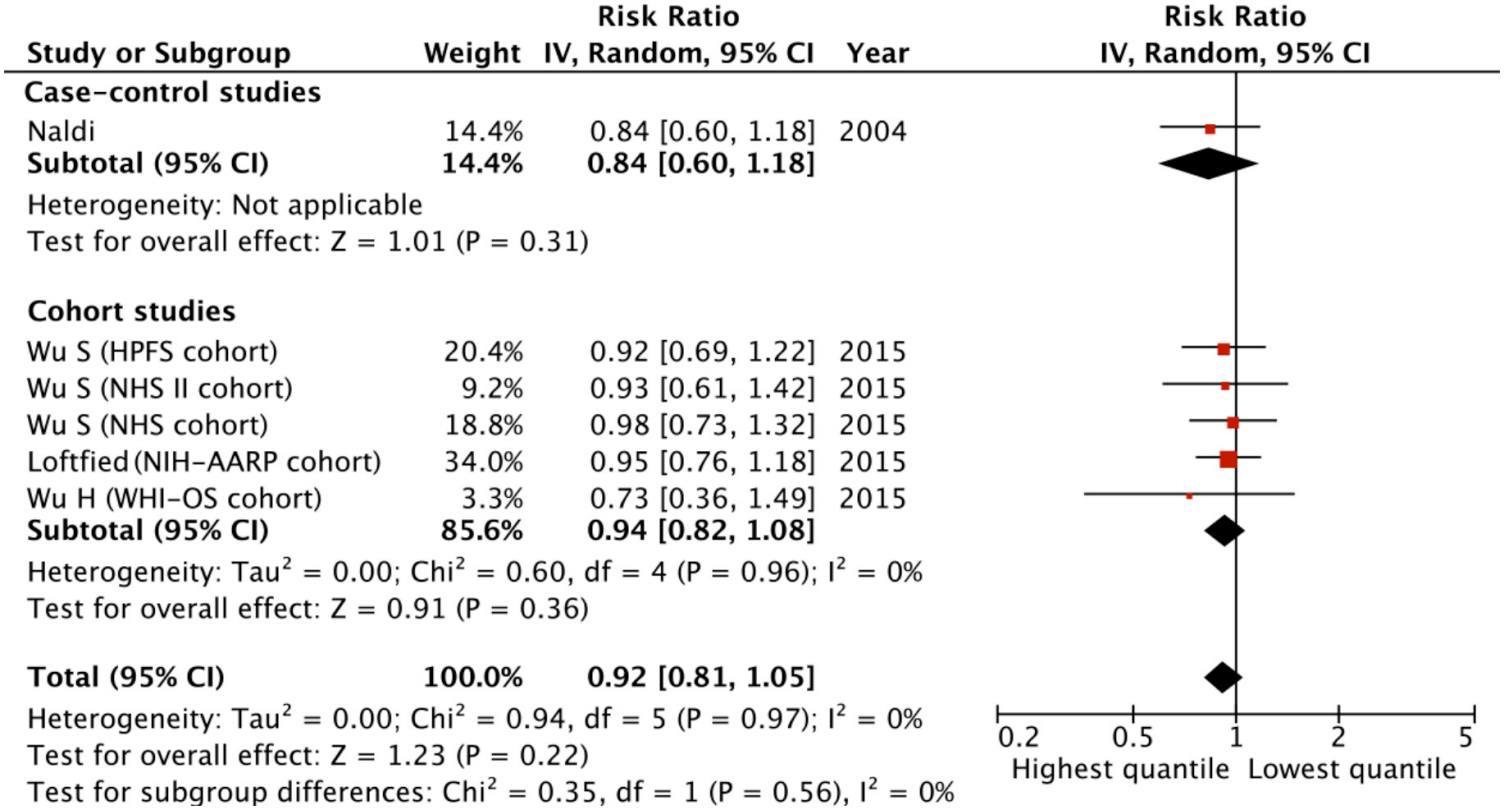
The relative risks (RRs) of melanoma for the highest versus lowest quantity of decaffeinated coffee intake in case-control studies and cohort studies. The square represents the study-specific RR and the size of the squares reflects the statistical weight in the meta-analysis; horizontal lines represent the 95% confidential intervals (95% CIs); the diamond indicates the overall RR with its 95% CI under the random-effects model.

Four studies (one case-control study and three cohort studies) with six subgroups have evaluated the association between decaffeinated coffee intake level and MM risk [9,12,13,16] (Table 2). The pooled RR was 0.92 (95% CI = 0.81–1.05) for those with highest versus lowest quantity of intake, suggested that decaffeinated coffee may not influence the MM risk (Fig 4). The Q-test (Q = 0.94, df = 5, P = 0.967) and the I^2^ (0%) statistic suggested that no significant heterogeneity between the studies was evident. None of the study-level characteristics significantly affected the results of the multiple studies as suggested by the meta-regression analyses (S3 Table). The sensitivity analyses also found no individual study that significantly affected the pooled estimate. No publication bias was noticed as suggested by the funnel plot (S2 Fig) and the Egger’s test (P = 0.109). We also found no significant association between the decaffeinated coffee intake level and the MM risk in any stratification studies by study design, participants’ gender, quality score or study region (Table 2).

## Discussion

Regular coffee contains numerous bioactive compounds including polyphenols, diterpenes, trigonelline and caffeine *etc*. Epidemiological studies have reported that coffee may have preventative effects against cardiovascular disease and cancer caused mortality [24], type 2 diabetes [25], stroke [26], prostate cancer [27] and endometrial cancer [28]. In contrast, higher coffee intake was found to be associated with increased risk for lung cancer [29], bladder cancer [30] and gastric cancer [31]. Compounds in coffee have been reported to have anticancer activities against UV-light induced skin carcinogenesis in experimental tests [32–34]. It was supposed that higher intake of coffee, which is rich in caffeine, might lead to decreased risk of MM. Here, we firstly evaluated the association between the coffee intake level and the MM risk through meta-analysis methods and found that participants with more caffeinated coffee intake may have reduced MM risk. In contrast, no significant association was noticed between decaffeinated coffee intake level and MM risk. These data further provided evidence for the chemo-preventive effects of caffeine against the MM risk.

There were several mechanisms for the cancer preventative effects of caffeine against MM. Animal studies have suggested that caffeine inhibits the UVB-induced thymine dimers formation and reduces sunburn lesions in the epidermis of experimental mice [35,36]. Topical application of caffeine enhanced the UVB-induced apoptosis, but it was inactive on non-UVB-treated normal skin in the hairless SKH-1 mice [37]. Oral administration pre-treatment of caffeine also increased the p53-positive cells, p21-positive cells and apoptotic sunburn cells in the epidermis [37]. Meanwhile, oral administration of coffee had similar stimulatory effects on UV-induced cellular apoptosis in the epidermis of SKH-1 mice [34]. In tumor cells, caffeine could lead to selective radiosensitization of p53-deficiency cells through activating the p34^cdc2^ kinase [38] and the radiosensitizing effects of the caffeine were also related to its inhibitory activities on the catalytic activity of both ATM and the related kinase, ATM and Rad3-related (ATR) [39]. Lu et al. found that administration of caffeine enhances the removal of DNA-damaged cells through inhibiting the ATR-mediated phosphorylation of Chk1 and prematurely increasing the number of cyclin B1-containing cells that undergo lethal mitosis [40]. Abnormal activity in the NF-kB signaling pathway is critical to the transformation of skin cells in the tumorigenesis, and caffeine is reported to have inhibitory activities on UV-mediated NF-kB activation in melanoma cells [41]. Caffeine also inhibited the lung metastasis and reduced the tumor growth of the melanoma cells [7,42]. Therefore, there it is biologically plausible that higher caffeine intake via coffee or tea may reduce the MM risk in certain populations.

Although we did not find a statistically inverse relationship between decaffeinated coffee intake level and the MM risk, we could not exclude the possibility that there were other potential anticancer ingredients in coffee besides the caffeine. The predominant chlorogenic acid in coffee, 5-O-caffeoylquinic acid and its metabolite caffeic acid have been reported to suppress the UVB-induced skin carcinogenesis by inhibiting the cyclooxygenase-2 (COX-2) expression [43] or directly inhibiting the ERK1/2 activities [32]. The cellular studies have revealed that the diterpenes cafestol and kahweol could induce the cellular apoptosis [44] and protest against the oxidative stress and DNA damage [45]. *In vivo* studies have found that topical administration of diterpenes inhibits the inflammation in epidermal cells of mice [5]. The nicotinic acid and nicotinamide generated from trigonelline in coffee roasting have protective roles against UVB-induced skin carcinogenesis in cellular models [46–48]. Considering the important role of caffeine in the suppression of tumorigenesis, the anticancer activities of decaffeinated coffee could be relatively lower and studies with larger sample size are warranted to reveal the anticancer activities of decaffeinated coffee.

Meta-regression studies suggested that no study-level characteristics including study design, study quality score, median or average following-up time, participant gender, or level of coffee intake significantly influence the heterogeneity between the studies. These factors may account for a small proportion of the heterogeneity between the studies. Other factors including the UV exposure level, the amount of the caffeine in the coffee, caffeine intake from other sources, the preparation method of coffee etc. may also contribute to the heterogeneity between the studies. In the stratification studies, we found the evidence for the protective effects of caffeinated coffee was more prominent in cohort studies than in case-control studies, which may be due to the relative higher quality of the cohort studies (S1 Table) and smaller sample size from case-control studies. Interestingly, we found significant protective effects against melanoma in women, but not men, regarding the use of caffeinated coffee. It has been suggested that men usually received less UV exposure than the women due to their different dressing styles, and the incidence of melanoma is higher for women. Moreover, men who have higher caffeinated coffee intake levels usually ingest higher levels of alcohol and/or with heavier smoking habits, both of which are associated with increased risk for melanoma. Thus, the protective roles of caffeinated coffee were not as apparent as in women for men.

There are several limitations for the current study. First, the ranges for coffee intake differed between the studies, which may lead to the heterogeneity of risk estimates between the groups. The dose-response analysis also found a significantly negative relationship between caffeinated coffee intake level and MM risk in which the RR was 0.96 (95% CI = 0.94–0.98) per 1 cup/day caffeinated coffee intake compared to non-drinkers. The coffee the participants consumed are typologically different both in preparation and in the coffee amount present in the cup, which may contribute to the heterogeneity between the studies. Second, all the studies were performed in the United States or European countries, so whether the associations noticed here could be externalized to other populations are still unknown. Larger prospective studies performed in other countries or populations are warranted. Third, the interactions between caffeinated coffee intake level and UV-light exposure level and their roles in the MM risk are largely unknown as caffeine reduced the UV irradiation induced DNA damage and enhanced UV induced cellular apoptosis. Fourth, in the meta-analysis of published studies, publication bias may lead to a biased false positive association as the small studies with null results may not yet published. Although we found no statistically significant publication bias here, we could not exclude the possibility that such bias existed. Last but not the least, the estimates from the individual studies was adjusted for different cofounding factors, which may also contribute to the heterogeneity between the studies and the interactions between the coffee intake level the cofounding factors in the melanoma risk were also hard to determine.

In conclusion, this meta-analysis found that caffeinated coffee intake was negatively associated with the MM risk, but not so for the intake of decaffeinated coffee. Considering the high prevalence of coffee consumption and the rising incidence rate of melanoma, our study may provide important public health implications in the prevention of MM. However, more studies, especially the intervention studies are warranted to fully elucidate the chemo-preventive effects of caffeinated coffee against MM.

## Supporting Information

S1 Fig Funnel plot of the association between higher caffeinated coffee intake and the melanoma risk compared to those of lower intake level for publication bias identification. (TIF)

S2 Fig Funnel plot of the association between higher decaffeinated coffee intake and the melanoma risk compared to those of lower intake level for publication bias identification. (TIF)

S1 File MOOSE Checklist for the meta-analysis study. (DOCX)

S2 File PRISMA Checklist for the meta-analysis. (DOC)

S1 Table The study quality for each subgroup studies according to the Newcastle-Ottawa quality scale. (DOCX)

S2 Table Univariate meta-regression analysis between the logrithm relative risk of melanoma for the highest vs. lowest quantile of caffeinated coffee intake and the basic characteristics of the study. (DOCX)

S3 Table Univariate meta-regression analysis between the logrithm relative risk of melanoma for the highest vs. lowest quantile of decaffeinated coffee intake and the basic characteristics of the study. (DOCX)

## Abbreviations

95% CI: 95% confidential interval
MM: malignant melanoma
OR: odds ratio
RR: relative risk
UV: ultra-violet
