# Supplementary figures and images for: Higher Caffeinated Coffee Intake Is Associated with Reduced Malignant Melanoma Risk: A Meta-Analysis Study
Source: PLoS One. 2016 Jan 27;11(1):e0147056. doi: 10.1371/journal.pone.0147056 (PMC4729676; doi:10.1371/journal.pone.0147056)

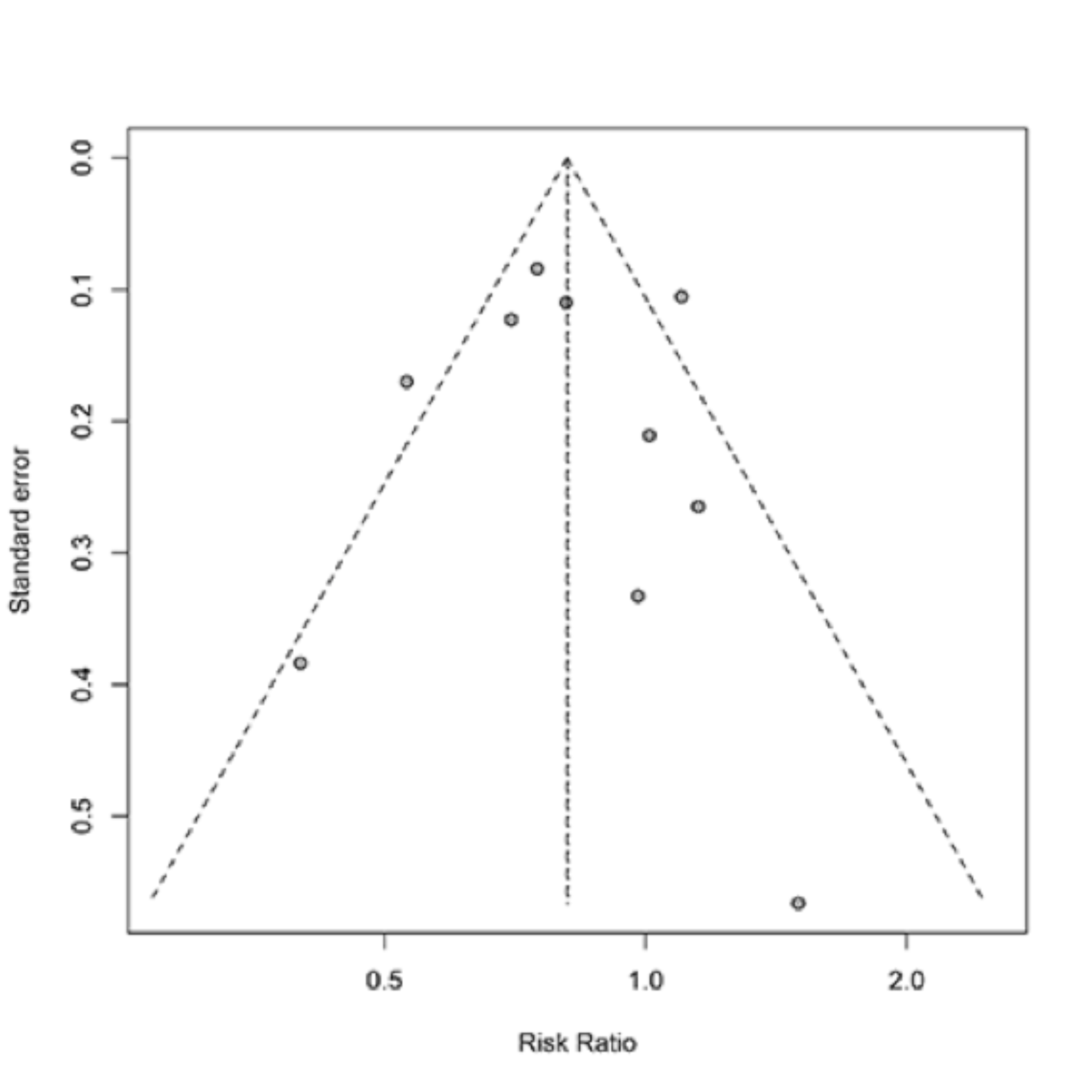

Supplement: S1 Fig — (TIF) [file pone.0147056.s001.tif]

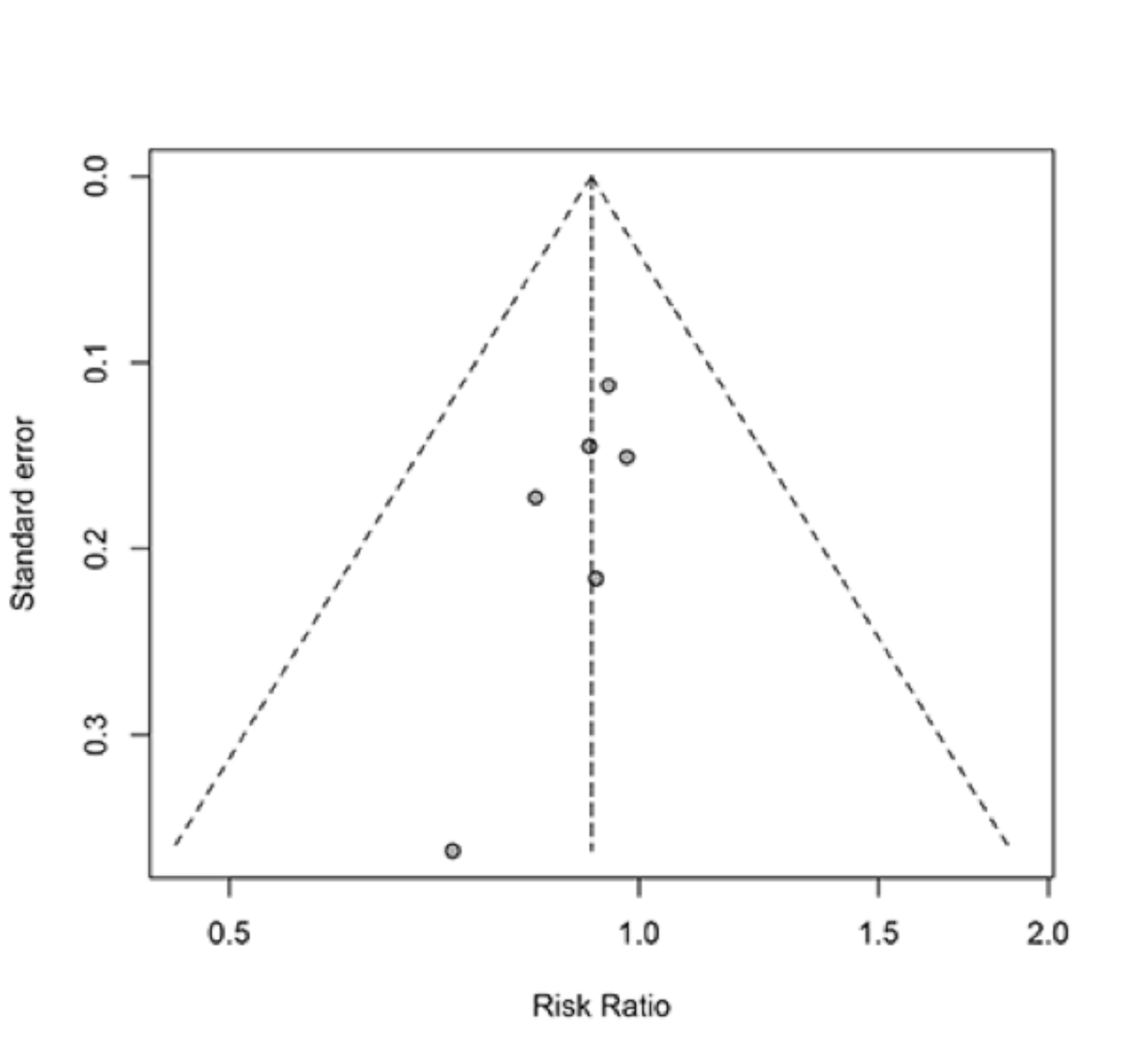

Supplement: S2 Fig — (TIF) [file pone.0147056.s002.tif]
