# Supplementary material for: Higher Caffeinated Coffee Intake Is Associated with Reduced Malignant Melanoma Risk: A Meta-Analysis Study
Source: PLoS One. 2016 Jan 27;11(1):e0147056. doi: 10.1371/journal.pone.0147056 (PMC4729676; doi:10.1371/journal.pone.0147056)
Supplement: S1 File — (DOCX) [file pone.0147056.s003.docx]

**S1 File. MOOSE Checklist**

**Higher caffeinated coffee intake is associated with reduced malignant melanoma risk: a meta-analysis study**

| **Criteria** | | **Brief description of how the criteria were handled in the meta-analysis** |
| --- | --- | --- |
| **Reporting of background should include** | |  |
| √ | Problem definition | Many previous epidemiological studies have investigated the associations between coffee intake level and melanoma risk; however, the findings were inconsistent. Herein we would like to conduct a systemic review of the association between the caffeinated and decaffeinated coffee intake level and the risks for melanoma. |
| √ | Hypothesis statement | Caffeinated coffee intake may have protective effects against melanoma. |
| √ | Description of study outcomes | Melanoma risk. |
| √ | Type of exposure or intervention used | The caffeinated or decaffeinated coffee intake level. |
| √ | Type of study designs used | We included case-control, cohort studies that have determined the association between coffee intake level and melanoma risk. |
| √ | Study population | We placed no restriction. |
| **Reporting of search strategy should include** | |  |
| √ | Qualifications of searchers | The credentials of the investigators are indicated in the author list. |
| √ | Search strategy, including time period included in the synthesis and keywords | PubMed from 1980 – November 2015  MEDLINE from 1980 –November 2015  See Figure 1 of the article |
| √ | Databases and registries searched | PubMed and MEDLINE. |
| √ | Search software used, name and version, including special features | No special software was used. However, EndNote was used to check the retrieved citations and eliminate duplications |
| √ | Use of hand searching | References were also checked to identify any missing studies. Details of the study were thoroughly examined in order to exclude potentially overlapping data. |
| √ | List of citations located and those excluded, including justifications | The detail literature research process was shown in Figure 1. |
| √ | Method of addressing articles published in languages other than English | Studies published in languages other than English was not included in the current study. |
| √ | Method of handling abstracts and unpublished studies | Abstracts were thoroughly reviewed. |
| √ | Description of any contact with authors | None. |
| **Reporting of methods should include** | |  |
| √ | Description of relevance or appropriateness of studies assembled for assessing the hypothesis to be tested | Detailed inclusion and exclusion criteria were described in the Figure 1. |
| √ | Rationale for the selection and coding of data | Data extracted from each of the studies were relevant to the population characteristics, study design, participants, number of participants, coffee intake level and corresponding risk estimates and the adjustments. |
| √ | Assessment of confounding | The risk estimates with the most completed adjustments in the individual studies were selected for pooling studies. |
| √ | Assessment of study quality, including blinding of quality assessors; stratification or regression on possible predictors of study results | We conducted sensitivity analysis to determine whether any individual study will affect the overall estimates. |
| √ | Assessment of heterogeneity | Heterogeneity of the studies was evaluated using the Cochrane’s Q test and I^2^ statistic. |
| √ | Description of statistical methods in sufficient detail to be replicated | The meta-analysis methods were described in the Materials and Methods parts. |
| √ | Provision of appropriate tables and graphics | The Figure 1 showed the flow chart of the selection procedures. The Figure 2 to 4 showed the results of the meta-analysis. Table 1 and 2 showed the details of the analysis results. |
| **Reporting of results should include** | |  |
| √ | Graph summarizing individual study estimates and overall estimate | Figure 2-4. |
| √ | Table giving descriptive information for each study included | Table 1. |
| √ | Results of sensitivity testing | In the results part. No single study was found to significantly affect the overall pooled results. |
| √ | Indication of statistical uncertainty of findings | 95% confidence intervals were presented for all summary estimates. The heterogeneity between the studies was evaluated and the publication bias was tested. |
| **Reporting of discussion should include** | |  |
| √ | Quantitative assessment of bias | Heterogeneity between the studies was evaluated and the publication bias was tested with the funnel plots and Egger’s tests. |
| √ | Justification for exclusion | Studies with overlapping studied populations were excluded from the study. |
| √ | Assessment of quality of included studies | We conducted the sensitivity analyses to identify the individual study that may affect the overall pooled data and the heterogeneity between the studies. |
| **Reporting of conclusions should include** | |  |
| √ | Consideration of alternative explanations for observed results | Caffeinated coffee have anticancer activities for melanoma risk but not for decaffeinated coffee, which suggested that caffeine may underlying such associations. The recall bias and selection bias for the included studies may influence the pooled estimates. These have been thoroughly discussed in the manuscript. |
| √ | Generalization of the conclusions | Our findings have revealed that caffeinated coffee may have chemo-preventive effects against melanoma risk. |
| √ | Guidelines for future research | Larger prospective studies and the intervention studies should be conducted to elucidate the preventive effects for caffeinated coffee on melanoma risk. |
| √ | Disclosure of funding source | Provided in the Acknowledgment part of manuscript. |
