# Supplementary material for: Higher Caffeinated Coffee Intake Is Associated with Reduced Malignant Melanoma Risk: A Meta-Analysis Study
Source: PLoS One. 2016 Jan 27;11(1):e0147056. doi: 10.1371/journal.pone.0147056 (PMC4729676; doi:10.1371/journal.pone.0147056)
Supplement: S1 Table — (DOCX) [file pone.0147056.s005.docx]

**S1 Table. The study quality for each subgroup studies according to the Newcastle-Ottawa quality scale**

| **Study** | **Study type** | **Reference**  **No.** | **Selection** | **Comparability** | **Exposure** | **Total score** |
| --- | --- | --- | --- | --- | --- | --- |
| Naldi, 2004 | Case-Control | 16 | 2 | 2 | 2 | 6 |
| Fortes, 2013 | Case-Control | 10 | 2 | 2 | 2 | 6 |
| Veierod, 1997 (Women) | Cohort | 11 | 3 | 2 | 2 | 7 |
| Veierod, 1997 (Men) | Cohort | 11 | 3 | 2 | 2 | 7 |
| Nilson, 2010 (VIP) | Cohort | 14 | 3 | 2 | 2 | 7 |
| Wu H, 2015 (WHI-OS) | Cohort | 12 | 2 | 2 | 1 | 5 |
| Wu S, 2015 (NHS II) | Cohort | 9 | 3 | 2 | 2 | 7 |
| Wu S, 2015 (NHS) | Cohort | 9 | 3 | 2 | 2 | 7 |
| Wu S, 2015 (HPS) | Cohort | 9 | 3 | 2 | 2 | 7 |
| Loftfield, 2015 (NIH-AARP) | Cohort | 13 | 3 | 2 | 2 | 7 |
