# Supplementary material for: Higher Caffeinated Coffee Intake Is Associated with Reduced Malignant Melanoma Risk: A Meta-Analysis Study
Source: PLoS One. 2016 Jan 27;11(1):e0147056. doi: 10.1371/journal.pone.0147056 (PMC4729676; doi:10.1371/journal.pone.0147056)
Supplement: S2 Table — (DOCX) [file pone.0147056.s006.docx]

**S2 Table. Univariate meta-regression analysis between the logrithm relative risk of melanoma for the highest vs. lowest quantile of caffeinated coffee intake and the basic characteristics of the study.**

| **Factor** | **β** | **Se(β)** | **P-value** |
| --- | --- | --- | --- |
| Study type (Cohort vs. Case-control) | 0.15 | 0.26 | 0.569 |
| Quality score | -0.04 | 0.16 | 0.787 |
| Median follow time (years) | 0.002 | 0.014 | 0.892 |
| Low boundary cutoff of highest caffeinated coffee intake category | 0.03 | 0.06 | 0.672 |
| Low boundary cutoff of lowest caffeinated coffee intake category | 0.08 | 0.44 | 0.857 |
| Sex (Women vs. Men) | -0.37 | 0.15 | 0.072 |
| Study region (USA vs. other) | -0.12 | 0.26 | 0.661 |
