# Supplementary material for: Higher Caffeinated Coffee Intake Is Associated with Reduced Malignant Melanoma Risk: A Meta-Analysis Study
Source: PLoS One. 2016 Jan 27;11(1):e0147056. doi: 10.1371/journal.pone.0147056 (PMC4729676; doi:10.1371/journal.pone.0147056)
Supplement: S3 Table — (DOCX) [file pone.0147056.s007.docx]

**S3 Table. Univariate meta-regression analysis between the logrithm relative risk of melanoma for the highest vs. lowest quantile of decaffeinated coffee intake and the basic characteristics of the study.**

| Factor | β | Se(β) | P-value |
| --- | --- | --- | --- |
| Study type (Cohort vs. Case-control) | 0.11 | 0.19 | 0.587 |
| Quality score | 0.13 | 0.14 | 0.411 |
| Median follow time (years) | 0.003 | 0.010 | 0.804 |
| Low boundary cutoff of highest caffeinated coffee intake category | -0.02 | 0.07 | 0.746 |
| Low boundary cutoff of lowest caffeinated coffee intake category | -0.29 | 0.34 | 0.443 |
| Sex (Women vs. Men) | -0.02 | 0.19 | 0.935 |
| Study region (USA vs. other) | 0.11 | 0.19 | 0.587 |
